# Anti-hepatocellular carcinoma properties of the anti-alcoholism drug disulfiram discovered to enzymatically inhibit the AMPK-related kinase SNARK *in vitro*

**DOI:** 10.18632/oncotarget.11820

**Published:** 2016-09-02

**Authors:** Kaku Goto, Naoya Kato, Raymond T. Chung

**Affiliations:** ^1^ The Advanced Clinical Research Center, The Institute of Medical Science, The University of Tokyo, Tokyo 108-8639, Japan; ^2^ Liver Center and Gastrointestinal Division, Department of Medicine, Massachusetts General Hospital, Harvard Medical School, Boston, MA 02114, USA

**Keywords:** disulfiram, Antabuse, SNARK, NUAK2, HCC

## Abstract

We recently described that the anti-apoptotic AMPK-related kinase, SNARK, promotes transforming growth factor (TGF)-β signaling in hepatocellular carcinoma (HCC) cells, as a potentially new therapeutic target. Here we explored FDA-approved drugs inhibiting the enzymatic activity of SNARK, using an *in vitro* luminescence kinase assay system. Interestingly, the long-used anti-alcoholism drug disulfiram (DSF), also known as Antabuse, emerged as the top hit. Enzymatic kinetics analyses revealed that DSF inhibited SNARK kinase activity in a noncompetitive manner to ATP or phosphosubstrates. Comparative *in vitro* analyses of DSF analogs indicated the significance of the disulfide bond-based molecular integrity for the kinase inhibition. DSF suppressed SNARK-promoted TGF-β signaling and demonstrated anti-HCC effects. The chemical and enzymatic findings herein reveal novel pharmacological effects of and use for DSF and its derivatives, and could be conducive to prevention and inhibition of liver fibrosis and HCC.

## INTRODUCTION

Protein kinases are critically involved in nearly all cellular functions encompassing metabolism, gene expression, proliferation, motility, and death. Their dysregulation, contributes to serious diseases such as cancer, hypertension, Parkinson's disease, and autoimmune diseases [1], which are attractive therapeutic targets [2]. In particular, AMP-activated protein kinase (AMPK) has been recognized as a key regulator of energy balance, related to type 2 diabetes and cancer [3], and AMPK-related kinases (ARKs) are also being increasingly recognized as modulators of cell dynamics and metabolism and observed to be tumor promoters though their physiological properties are not yet well understood [4].

Liver diseases including fibrosis and consequential hepatocellular carcinoma (HCC) remain an important health menace [5], in urgent need of improved methods for prophylactic and therapeutic management [6]. We recently reported that sucrose-non-fermenting protein kinase 1 (SNF1)/AMPK-related protein kinase (SNARK), the fourth member of 14 ARKs [7], supported replication of hepatitis C virus (HCV), a causative agent of HCC, in genome-wide RNAi screen [8], and enhanced profibrogenic transforming growth factor (TGF)-β signaling in HCC cells [9], suggesting SNARK as an attractive pharmacological target. Hence we here sought to find repositionable approved drugs [10] inhibiting SNARK kinase activity implementing an *in vitro* luminescence kinase assay system. Interestingly, the top hit in an FDA-approved drug library was disulfiram (DSF), the long-used anti-alcoholism drug also known as Antabuse. Enzymatic kinetics analyses revealed that DSF inhibited SNARK kinase activity in a noncompetitive manner to ATP or phosphosubstrates. While a close DSF analog tetramethylthiuram disulfide (TR) exerted inhibition, its monosulfide analog tetramethylthiuram monosulfide (TMTM) did not, indicating the significance of the disulfide bond-based molecular integrity of DSF for the kinase inhibition. DSF was indeed proved in cell culture to suppress SNARK-enhanced TGF-β signaling monitored through plasminogen activator inhibitor (PAI)-1 activity. DSF also exhibited anti-HCC effects, to which HCC cells were conferred resistance by excessive supply of SNARK. The successful discovery of a SNARK inhibitor here discloses a novel mode to explain the recently recognized anti-cancer effects and anti-fibrogenic potential of DSF [11, 12]. These chemical and enzymatic findings will be conducive to prevention and inhibition of liver fibrosis and HCC.

## RESULTS

### Screen for SNARK kinase inhibitors *in vitro*

**Figure 1:**
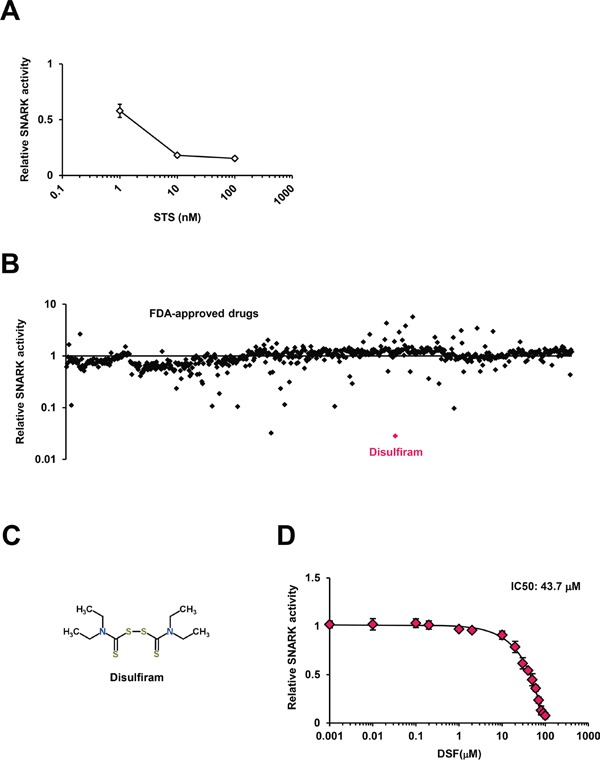
The SNARK kinase inhibitor screen *in vitro* **A.** The *in vitro* SNARK kinase assay system was validated using the known multi-kinase inhibitor STS as the control. **B.** The kinase assay was performed for 636 compounds in the FDA-Approved Drug Screen-well Library. Z scores of relative kinase activities in the presence of individual drugs compared with DMSO controls are indicated. **C.** The chemical structure of DSF retrieved from the database ChemSpider. **D.** The kinase inhibitory effects of DSF were examined at 0.001, 0.01, 0.1, 0.2, 1, 2, 10, 20, 30, 40, 50, 60, 70, 80, 90, and 100 μM *in vitro*.

To confirm the validity of the *in vitro* SNARK kinase assay system, which reports the amount of ATP consumed for phosphorylation as luminescent signals, we first tested the effects of staurosporine (STS), a known multi-protein kinase inhibitor [13], and consequently observed significantly decreased luminescence by STS (Figure 1A), authenticating the monitoring method. Next we implemented the kinase assay for 636 compounds in the FDA-Approved Drug Screen-well Library (Figure 1B); only three compounds demonstrated inhibition more than 90% in the primary screen and the top hit with around 97% inhibition was DSF, the long-used anti-alcoholism drug also known as Antabuse (Figure 1C). DSF was structurally independent from other hits with inhibition more than 50% (Supplementary Figure S1A and S1B). The robust inhibition by DSF more potent than other high-ranking candidates was validated separately, with the dose-dependent effects giving 50% inhibitory concentration (IC_50_) of 43.7 μM *in vitro* (Figure 1D), while luciferase activity itself was not suppressed by DSF (Supplementary Figure S1C) in agreement with the previous counter-screen [14].

### Kinetics and modes of SNARK kinase inhibition by DSF

**Figure 2:**
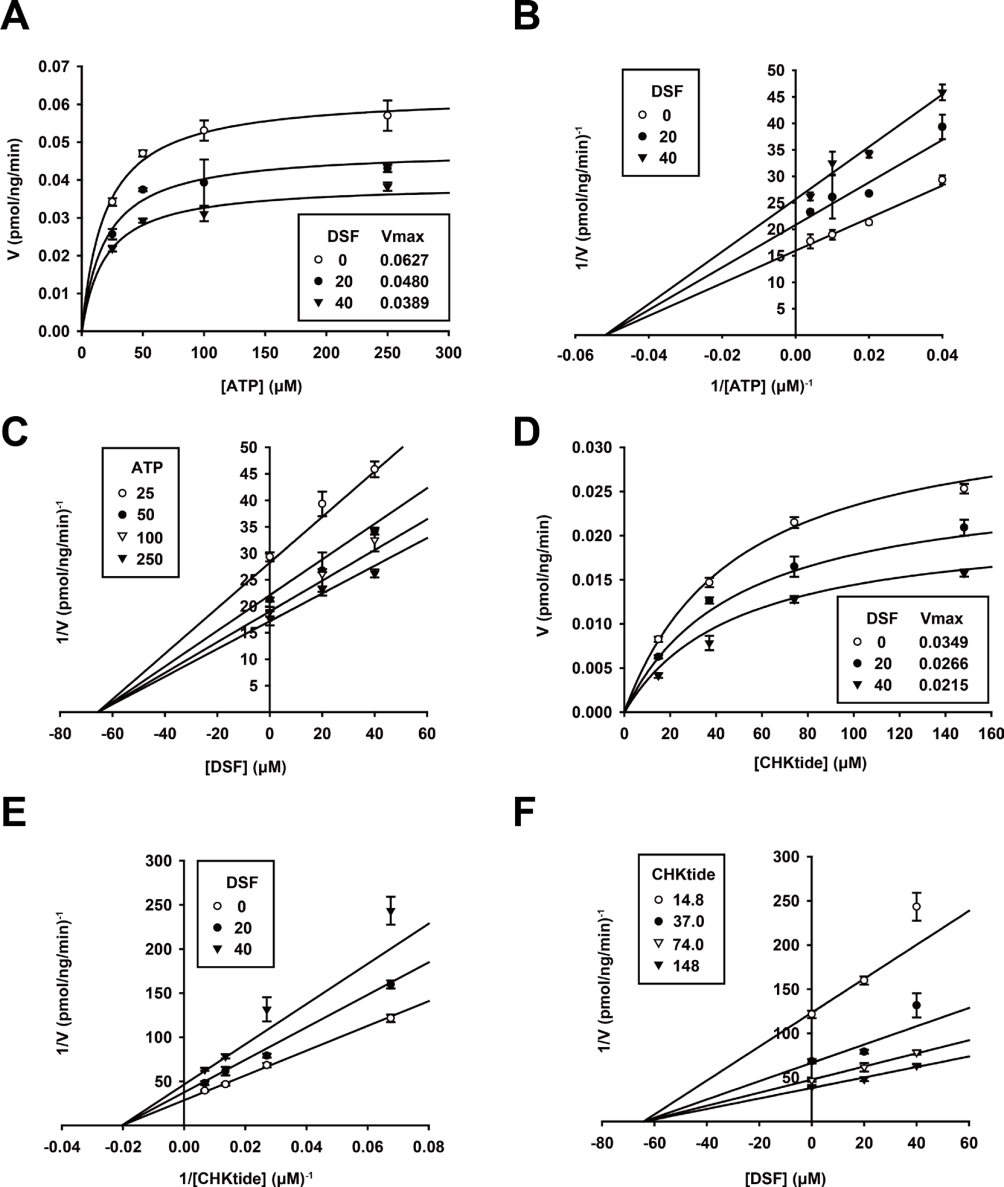
Enzymatic kinetics of SNARK kinase inhibition by DSF The *in vitro* SNARK kinase assay was performed with 25, 50, 100, and 250 μM ATP or 14.8, 37.0, 74.0, and 148 μM CHKtide in the presence of 0, 20, and 40 μM DSF. Subsequently effects of DSF on enzymatic kinetics of SNARK were calculated, yielding Michaelis-Menten plots **A.** and **D.** Lineweaver-Burk plots **B.** and **E.** and Dixon plots **C.** and **F.**

In order to clarify the mode of SNARK inhibition by DSF, enzymatic kinetics in the presence of DSF were investigated. The Michaelis-Menten plots of the kinase reaction with various concentrations of ATP (Figure 2A) implied non-competitive inhibition against ATP, which was confirmed by the Lineweaver-Burk and Dixon plots (Figure 2B and 2C, K_m_ = 19.3 ± 2.18 μM, K_i_ = 65.7 ± 6.09 μM). In addition, the reaction was also examined with various concentrations of the phosphosubstrate peptide CHKtide, whose kinetics are shown in the Michaelis-Menten plots (Figure 2D), with K_m_ = 48.9 ± 4.23 μM. The Lineweaver-Burk and Dixon plots revealed non-competitive inhibition against the phosphosubstrates (Figure 2E and 2F, K_i_ = 64.3 ± 5.17 μM), confirmed by no observation of competitive inhibition after the preincubation of the kinase with DSF either (Supplementary Figure S2).

**Figure 3:**
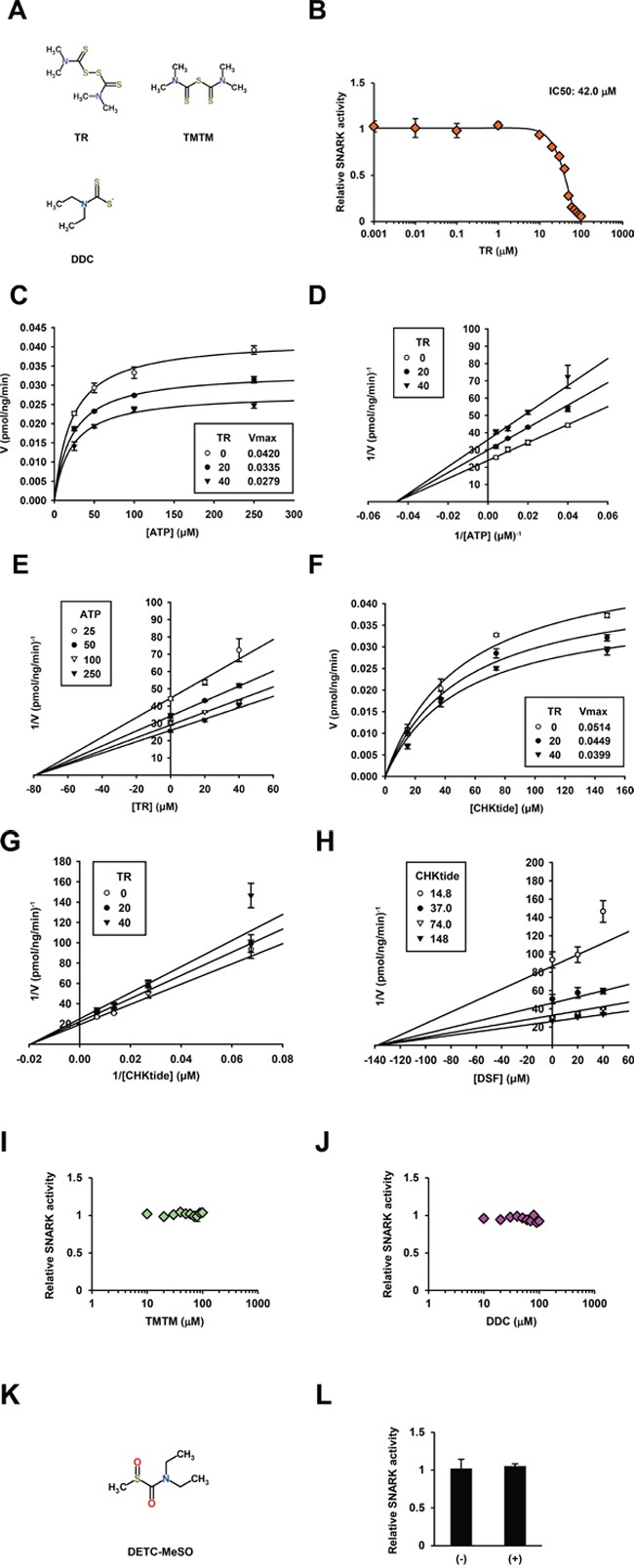
DSF analogs and enzymatic kinetics of SNARK kinase inhibition by TR **A.** Chemical structures of TR, TMTM, and DDC were retrieved from ChemSpider. **B.** Similarly to Figure 1D, the kinase inhibitory effects of TR were examined at 0.001, 0.01, 0.1, 1, 10, 20, 30, 40, 50, 60, 70, 80, 90, and 100 μM *in vitro*. Similarly to Figure 2, the *in vitro* SNARK kinase assay was performed with 25, 50, 100, and 250 μM ATP or 14.8, 37.0, 74.0, and 148 μM CHKtide in the presence of 0, 20, and 40 μM TR. Subsequently effects of TR on enzymatic kinetics of SNARK were calculated, yielding Michaelis-Menten plots. **C.** and **F.** Lineweaver-Burk plots **D.** and **G.** and Dixon plots **E.** and **H.** Similarly to (B), effects of TMTM and DDC, and DETC-MeSO **K.** on SNARK kinase activity were examined at 10, 20, 30, 40, 50, 60, 70, 80, 90, and 100 μM **I.** and **J.** and 100 μM **L.** respectively.

Next, to address the question of the functional groups responsible for the inhibition, the effects of the close DSF analogs TR and TMTM (Figure 3A), on the kinase reaction were tested; in actuality TR inhibited the kinase activity with the IC_50_ 42.0 μM (Figure 3B), and the Michaelis-Menten (Figure 3C), Lineweaver-Burk (Figure 3D), and Dixon (Figure 3E) plots (K_m_ = 21.8 ± 1.54 μM, and K_i_ = 79.0 ± 5.25 μM) suggested ATP-noncompetitive inhibition. Again kinase assays at various CHKtide concentrations produced the Michaelis-Menten (Figure 3F), Lineweaver-Burk (Figure 3G), and Dixon (Figure 3H) plots (K_m_ = 51.2 ± 5.00 μM, K_i_ = 138.0 ± 21.3 μM), indicating phosphosubstrate-noncompetitive inhibition. Meanwhile TMTM exhibited almost no inhibitory activities (Figure 3I) up to 1000 μM (Supplementary Figure S3A) and hence the disulfide bond was exhibited *in vitro* to be important for DSF to inhibit the SNARK kinase activity. With respect to the DSF metabolite diethyldithiocarbamate (DDC) (Figure 3A) [15], no inhibitory effects were displayed (Figure 3J) while only slight inhibition was observed at higher concentrations than 100 μM (Supplementary Figure S3B), and neither did S-Methyl-N, N-diethylthiocarbamoyl sulfoxide (DETC-MeSO) (Figure 3K), a further metabolite downstream of DDC and the known inhibitor of aldehyde dehydrogenase [15], inhibit the kinase activity *in vitro* up to 100 μM (Figure 3L) with mild inhibition at higher concentrations (Supplementary Figure S3C), wherefore biochemical significance of the DSF structure was confirmed. Thus enzymatic kinetics and chemical structure analyses uncovered the ATP- and phosphosubstrate-noncompetitive modes of SNARK inhibition by DSF via disulfide bond-based molecular integrity (Supplementary Table S1).

### Inhibition of SNARK-promoted TGF-β signaling by DSF

**Figure 4:**
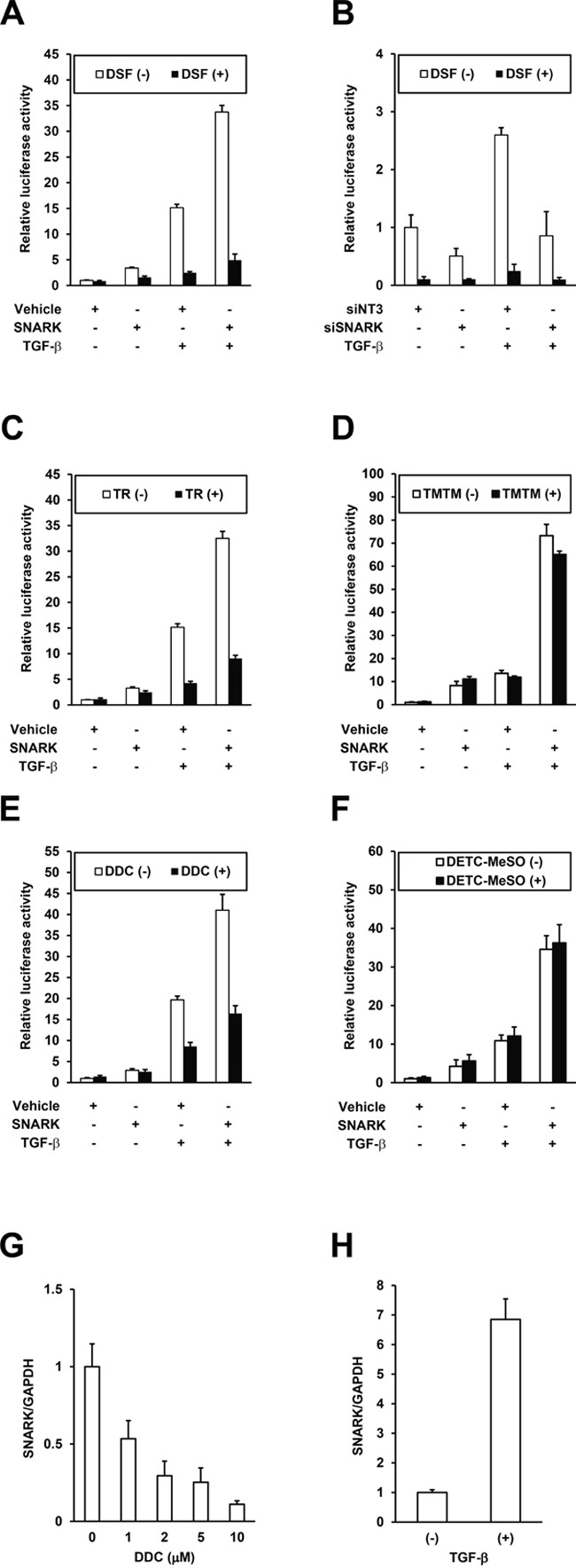
Inhibition of SNARK-promoted TGF-β signaling by DSF **A.** HepG2 cells were transfected with PAI/L and pRL-TK in combination with pEBMulti-Puro (Vehicle) or pEBMP-SNARK-3×FL (SNARK), followed by the treatment with DSF at 15 μM and TGF-β at 10 ng/mL 24 h later. On the next day the cells were lysed and the firefly and *Renilla* luciferase activities were measured. **B.** HepG2 cells were transfected with non-targeting siRNAs (siNT3) or siRNAs to SNARK (siSNARK), followed by transfection with PAI/L and pRL-TK 24h later. On the next day DSF at 10 μM and TGF-β at 10 ng/mL were added and 24h later the cells were lysed and the firefly and *Renilla* luciferase activities were measured. Similarly to (A), the assays were performed using TR **C.** TMTM **D.** DDC. **E.** and DETC-MeSO **F.** in place of DSF. Total RNA was extracted from HepG2 cells treated with DDC at indicated concentrations **G.** or 10 ng/mL TGF-β **H.** for 48h and SNARK mRNA levels were quantified by qRT-PCR with normalization to *GAPDH*. n ≥ 3. Error bars = SD.

As we recently reported that SNARK enhanced TGF-β signaling in HCC cells and the pharmacological inhibition of SNARK resulted in the suppression of profibrogenic signaling [9], the effects of DSF were next assessed in cell culture. The activity of a plasmid PAI/L encoding a luciferase reporter gene driven by promoter sequences of PAI-1, a transcriptional target of TGF-β [16], was enhanced by SNARK overexpression and the effects were further highlighted by TGF-β in HepG2 cells (Figure 4A, white bars) in accordance with our previous data [9]. DSF suppressed the SNARK-promoted luciferase activities in the absence and particularly presence of TGF-β (Figure 4A, black bars), endorsing the inhibitory efficacy *in vitro*, while no inhibition of luciferase activity itself by DSF was confirmed in HepG2 cells with the overexpression of luciferase protein (Supplementary Figure S4A). In turn, RNAi-mediated knockdown of SNARK expression decreased PAI/L activity whether treated with TGF-β or not (Figure 4B, white bars) consistent with past report [9], and the suppressive effects by DSF on PAI/L were relatively blunted (Figure 4B, black bars), indicating the dependence of the DSF-mediated TGF-β signaling inhibition on SNARK. Subsequently, the DSF analogs TR and TMTM with and without SNARK kinase inhibitory activity *in vitro*, respectively, were tested in the same setting. TR inhibited the SNARK-enhanced PAI/L activity, demonstrating all the more pronounced effects in the presence of TGF-β (Figure 4C), while TMTM did not affect PAI/L significantly (Figure 4D). Intriguingly, DDC, a key metabolite of DSF, decreased the PAI/L activity as well in the presence of TGF-β (Figure 4E) unlike DETC-MeSO (Figure 4F) though the both lacked the kinase inhibitory properties *in vitro* (Figure 3J and 3L). Therefore, possibly responsible factors such as other DDC metabolites than DETC-MeSO and/or intracellular biological effects of DDC without excluding SNARK-independent mechanisms are implied. Noteworthily observed, on the other hand, was the suppression of SNARK mRNA levels in HepG2 cells treated by DDC (Figure 4G), which could affect SNARK-stimulated cell biological phenotypes as TGF-β was found to upregulate SNARK expression levels (Figure 4H), though such pronounced effects were not directly evoked by the treatment with DSF and TR (Supplementary Figure S4B and S4C). Additionally, the replication of the profibrogenic virus HCV, dependent on SNARK [9], was also abrogated by DSF (Supplementary Figure S4D), which was supported by a recent report using HCV replicon cells [17]. Overall, DSF's inhibition of the *in vitro* kinase activity results in its suppression of SNARK-dependent profibrogenic signaling in HCC cells.

### Inhibition of SNARK-promoted HCC cell proliferation by DSF

**Figure 5:**
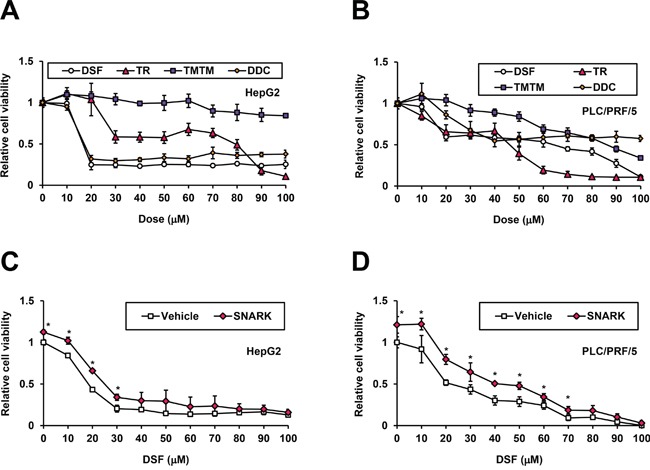
Inhibition of SNARK-promoted HCC cell proliferation by DSF HepG2 **A.** and PLC/PRF/5 **B.** cells were treated with DSF, TR, TMTM, and DDC at the indicated doses for 24 hours and the relative cell viabilities to untreated control cells were measured by CCK-8 assay. pEBMulti-Puro (Vehicle) or pEBMP-SNARK-3×FL (SNARK) was transfected into HepG2 **C.** or PLC/PRF/5 **D.** cells, followed by DSF treatment at the indicated concentrations 48h later. On the next day the relative cell viabilities to the control samples untreated and transfected with pEBMulti-Puro were measured similarly to (A) and (B). *P < 0.05 by Student's t-test. n = 4. Error bars = SD.

SNARK has been found to operate against apoptosis, functioning as a tumor promoter [18, 19]. Meanwhile, the anti-cancer effects of DSF have been described recently [12]. Hence we examined anti-HCC effects of DSF and presumed participation of SNARK in this mode of action. In HepG2 cells treated for 48h (Figure 5A), DSF markedly inhibited HCC cell growth at 20 μM as did TR relatively moderately, in contrast to TMTM exerting no effects at even higher concentrations, in proportion to its *in vitro* kinase inhibitory activity. DDC exerted pronounced anti-proliferative effects similarly to DSF though DDC itself was known to possess anti-cancer activities [20] independently. Correspondingly, remarkable cytotoxicities were caused by DSF and DDC while TR and TMTM exerted milder and no cytotoxicities, respectively (Supplementary Figure S5A). In the other liver cancer cell line PLC/PRF/5, generally the effects of DSF and TR were stronger than those of TMTM, though DDC exhibited a unique profile (Figure 5B). These data suggest that DSF and TR demonstrate stronger cytotoxicity, particularly at higher doses (Supplementary Figure S5B), while Huh7 cells are highly susceptible to DSF (Supplementary Figure S5C), being inhibited by about 90% even at 10 μM, below the concentrations used for the cell-based assays in HepG2 and PLC/PRF/5 cells. Meanwhile, the levels of cell proliferation (Supplementary Figure S5D) and cytotoxicity (Supplementary Figure S5E) of PXB cells, normal human hepatocytes isolated from chimeric mice with a humanized liver (PXB mice) [21], were minimally altered, supporting the liver cancer cell-specific toxicity of DSF.

To determine whether SNARK was involved in the anti-HCC effects by DSF, we next conducted the assay in the presence of SNARK overexpression. Both HepG2 and PLC/PRF/5 cells were conferred resistance to DSF at lower concentrations (Figure 5C and 5D) by the excessive supply of SNARK (Supplementary Figure S5F), which was not observed with the kinase-dead K81M and unphosphorylated T208A mutants [9] in HepG2 cells (Supplementary Figure S5G) and PLC/PRF/5 cells (Supplementary Figure S5H), confirming SNARK as a tumor promoter and a pharmacological target. Taken together, DSF exhibited anti-cancer effects against HCC as well, through its actions on SNARK.

## DISCUSSION

Growing evidence has demonstrated that SNARK possesses anti-apoptotic properties [18, 19] and is involved in cancer [22–24]. We recently reported the novel feature of SNARK as a profibrogenic factor in HCC cells through the promotion of TGF-β signaling [9]. Therefore the successful identification of DSF to inhibit SNARK kinase offers real opportunity to exploit the drug and dissect the mechanisms for further pharmacological development. An understanding of the basic enzymatic and biochemical properties of the SNARK-DSF interaction is important.

The enzymatic kinetics analyses suggested that DSF is an ATP-noncompetitive inhibitor (Figure 2), implying that DSF could target a regulatory site(s) for ATP hydrolysis rather than compete at the ATP-binding site. Also noncompetitively inhibited was CHKtide, connoting that a modulatory domain distinct from the catalytic center is the DSF target. It is very interesting to speculate that an identical regulatory site could affect both ATP and CHKtide reactions, though behaviors of actual proteins catalyzed in cells may not be entirely reflected by CHKtides. The identification of potential DSF-binding targets of SNARK is of great interest; the close DSF analog TR shared a similar 3D structure with and retained the *in vitro* kinase inhibitory activity in the same manner as DSF. However, TMTM, the monosulfide analog of TR, was conformationally distinct from DSF and TR and devoid of the kinase inhibition. The disulfide bond-based structure is assumed to be fit for DSF docking with the kinase, and accordingly further structural analyses including crystallography of SNARK and its complex with DSF are warranted based on the insights from DSF and its analogs as chemical probes. This could stimulate rational drug development through the well-known characteristic of DSF.

In our assays, SNARK was shown to promote TGF-β signaling in HCC cells, which was suppressed by the SNARK inhibitor DSF as expected. Intriguingly, a recent study reported that DSF inhibited TGF-β-induced epithelial-mesenchymal transition in breast cancer cells [11] in conformity with the downregulation of TGF-β signaling by DSF in zebra fish cells [25]. Furthermore, fibrosis-attenuating properties of DSF were observed in a mouse model of non-alcoholic steatohepatitis [26]. Therefore SNARK has been projected as the target of anti-fibrogenic effects through TGF-β signaling by DSF. As for DDC, a DSF metabolite, though it lacks SNARK kinase inhibition *in vitro*, suppressive effects on PAI/L when treated with TGF-β were still detected, potentially indicating intracellular circumstance-specific suppression of TGF-β signaling. Of interest, DDC, but not DSF (Supplementary Figure S4B), was found to decrease the SNARK expression levels (Figure 4G), which was in actuality elevated by TGF-β (Figure 4H). This could at least partly explain the retained suppressive effects of DDC particularly in the presence of TGF-β, and denoted the effectiveness of breaking the vicious circle of reciprocal upregulation between SNARK and TGF-β. The enzymatic and expression inhibition of SNARK by DSF and its metabolite DDC, respectively, are thus postulated to accentuate the potency of DSF coordinately, and further detailed mechanistic studies are underway to decipher these findings.

The noted function of SNARK as a tumor promoter with anti-apoptotic properties was indeed verified in HCC cells through observation of their resistance to the cytotoxic effects of DSF conferred by the excessive supply of SNARK. In recent years, mounting evidence suggests anti-cancer properties of DSF across cancer types [12], and HCC cells are no exception; DSF exerted anti-HCC effects through inhibition of hypoxia-induced gene expression and hypoxia-inducible factor activity for tumor adaptation to hypoxia [27]. Also tumorigenicity of tumor-initiating HCC cells was impaired via reactive oxygen spieces-p38 pathway and *Glypican 3* by DSF [28]. Another recent study showed DSF, in Hep3B cells, triggered apoptosis intrinsically and extrinsically, including activation of the pathway through death receptor CD95 [29], through which SNARK reportedly rendered breast cancer cells apoptosis-resistant and invasive [18]. Such modes of anti-cancer actions by DSF as above allude to critical molecules associated with and potentially phosphorylated as substrates by SNARK, whose unknown functions attract much attention for reciprocal elucidation between physiological roles and therapeutic implications. Again DDC, with anti-cancer effects via chelating into metal ions [20] and reported to significantly improve the overall survival in breast cancer patients [30], demonstrated unique profiles of cytotoxicity to HCC cells, and indicated the effects by DSF are to be kept even after being metabolized at least reducing SNARK expression (Figure 4G); lately further improvement in stable delivery and anti-cancer effects of DDC was reported *in vivo* [31]. Utilization of intact DSF, simultaneously, is being investigated for its anti-cancer properties, and such a method was recently developed by nanocapsule-protected medicine [32], achieving favorable anti-cancer effects. Still the cytotoxic efficacy could fluctuate depending on individual cell types with various genetic and pathoetiological backgrounds as was the case with PLC/PRF/5 cells positive for hepatitis B virus proteins (Figure 4B) and anti-cancer agents [33]. Thus, together with the observation of suppressive effects on TGF-β signaling, leading to the inhibition of cancer cell metastasis and tumor growth *in vivo* [11], DSF is thought to be an effective therapeutic agent for solid tumors including HCC.

In practice, therapeutic effects of DSF have been observed in metastatic non-small cell lung cancer (NSCLC) patients with an increase in survival [34] and solid tumors [35], and also is under clinical trials for glioblastoma [12, 36] and advanced HCC (UMIN000008529, http://www.umin.ac.jp/) in expectation of exploitation as a chemotherapeutic agent. Another attractive feature of DSF is the tolerability and safety demonstrated over years of clinical experience with a large number of patients, and also in the NSCLC patients [34]. Plasma concentrations of DSF in several reports [37, 38], reaching even above 100 μM, routinely exceeded the IC_50_ of the *in vitro* kinase assay described here, so SNARK inhibitory concentrations are expected to be achieved safely in patients. It should be cautioned that the possible novel clinical use of DSF would be in persons with chronic liver disease at risk for cirrhosis and HCC, and understandably toxicity would need to be carefully monitored especially in persons with advanced cirrhosis, as summarized in disease guidelines [39]. However, DSF has long been used safely in patients who often harbor significant alcoholic liver disease.

Here the enzymatic inhibition of SNARK and resultant anti-TGF-β/HCC effects by DSF provide us with new biochemical and pragmatic insights. The utility and a newly revealed property of DSF were mechanistically suggested and in turn the significance of regulatory sites for SNARK kinase activity as validated drug target can now be comprehensively assessed using DSF as a chemical probe. Also DSF derivatives with better potencies could be examined [40] in addition to devising improved methods of delivery [32, 41]. Further chemical and biological analyses would accelerate understanding of SNARK functions and at the same time the chemical search for and development of potent SNARK inhibitors continue. Finally well-tolerated and more effective regimens for HCC management can be expected to be developed using DSF and its analogs/derivatives, inspired and reinforced by the discovery in this study.

## MATERIALS AND METHODS

### Compounds and cells

DSF, and STS, TR, TMTM and Sodium diethyldithiocarbamate trihydrate were purchased from Selleckchem (Houston, TX) and Sigma-Aldrich (St. Louis, MO), respectively. DETC-MeSO was purchased from Santa Cruz Biotechnology (Santa Cruz, CA). TGF-β was purchased from R&D (Minneapolis, MN). Antibodies to luciferase, HCV NS3, and FLAG and β-actin (ACTB) were purchased from MBL (Aichi, Japan), Abcam (Cambridge, United Kingdom), and Sigma-Aldrich, respectively. The FDA-Approved Drug Screen-well Library and Cell Counting Kit (CCK)-8 were obtained from Enzo Life Sciences (Farmingdale, NY) and Dojindo (Kumamoto, Japan), respectively. Cytotoxicities of compounds were determined with the LDH cytotoxicity detection kit (Takara Bio, Shiga, Japan). HepG2, PLC/PRF/5, and Huh7 cells were cultured according to the protocols of American Type Culture Collection (Manassas, VA) and Japanese Collection of Research Bioresources Cell Bank (Osaka, Japan). The cell lines were authenticated by short tandem repeat method (Bex, Tokyo, Japan) in Jan 2016. PXB cells were purchased from Phoenix Bio (Hiroshima, Japan). JFH1 virus infection was performed as described previously [9].

### Plasmid

pCMV14-SNARK_NCBI-3×FL was constructed by primers EcoRI-SNARK-F 5′-TTGAATTCGGCCACCATGGAGTCGCTGGTTTTC-3′, XbaI-SNARK-ΔTGA-R 5′-AATTCTAGAGGTGAGCTTTGAGCAGAC-3′, SNARK-1323-G-F 5′-GGCAGGCTGCCCCGCTGCTC-3′, and SNARK-1323-G-R 5′-GAGCAGCGGGGCAGCCTGCC-3′, using pCMV14-SNARK-3×FL as a template [9]. Sequences containing SNARK open reading frame were amplified by the primers XhoI- SNARK-F 5′-ATTCTCGAGGGCCACCATGGAGTCGCTGGTTTTC-3′ and NotI-SNARK-3×FL-R 5′-AATGCGGCCGCCTACTTGTCATCGTCATCCTTG-3′ from pCMV14- SNARK_NCBI-3×FL, and subcloned into pEBMulti-Puro (Wako, Osaka, Japan), producing the expression plasmid pEBMP-SNARK-3×FL. Similarly, pCMV14-SNARK(K81M)_NCBI-3×FL and pCMV14-SNARK(T208A)_NCBI-3×FL were generated by primers EcoRI-SNARK-F, XbaI-SNARK-ΔTGA-R, SNARK-1323-G-F and SNARK-1323-G-R using pCMV14-SNARK(K81M)-3×FL and pCMV14-SNARK(T208A)-3×FL as templates, respectively [9]; subsequently using these constructs as templates, pEBMP-SNARK(K81M)-3×FL and pEBMP-SNARK(T208A)-3×FL were produced by primers EcoRI-SNARK-F and NotI-SNARK-3×FL-R. pEBMP-Luc was constructed by primers XhoI-Luc-F 5′-ATTCTCGAGGGCCACCATGGAAGACGCCAAAAACATAAAGAAAGGC-3′ and NotI-Luc-R AATGCGGCCGCTTACAATTTGGACTTTCCGCCCTTCTTGGC using PAI/L as a template.

### *In vitro* kinase assay

NUAK2 Kinase Enzyme System was purchased from Promega (Madison, WI). Kinase assay was performed according to the manufacturer's protocol and the luminescence was monitored (n = 4). Briefly, 12.5 ng of recombinant SNARK was incubated with CHKtide and ATP, in the presence or absence of DMSO or compounds, for 60 min at room temperature, followed by ATP depletion and subsequent detection of converted ADP via luminescence.

### Enzyme kinetic analysis

The mode of enzymatic inhibition was calculated bySigmaplot (Systat Software, San Jose, CA), with the best fit models determined by the Akaike Information Criterion.

### Luciferase assay

Firefly luciferase activity was monitored by a dual-luciferase reporter assay system (Promega) and normalized to *Renilla* luciferase activities from pRL-TK as described previously [9]. SiNT3 (#D-001210-03) and siSNARK (SI02660224) were purchased from GE Healthcare Bio-Sciences (Pittsburgh, PA) and QIAGEN (Valencia, CA), respectively, and the assay was performed as described previously [9], with 3 pmol siRNAs at 5 nM.

### Quantitative reverse transcription-polymerase chain reaction

Relative mRNA and viral RNA levels were quantified as previously described [9] using the following primer sets: SNARK-F 5′-GATGCACATACGGAGGGAGAT-3′ and SNARK-R 5′-GCTGGCATACTCCATGACGAT-3′ for SNARK, JFH1-F 5′-CTGTCTTCACGCAGAAAGCG-3′ and JFH1-R 5′- TCGCAACCCAACGCTACTCG-3′ and GAPDH-F2 5′-AAGGTGAAGGTCGGAGTCAAC-3′ and GAPDH-R2 5′-GGGGTCATTGATGGCAACAATA-3′ for glyceraldehyde-3-phosphate dehydrogenase (GAPDH), with the value of *SNARK* and *JFH1* normalized to that of *GAPDH*.

### Western blotting

Total protein was resolved by SDS-PAGE and subjected to western blotting as described previously [9].

## SUPPLEMENTARY FIGURES AND TABLE


