# Supplementary material for: Anti-hepatocellular carcinoma properties of the anti-alcoholism drug disulfiram discovered to enzymatically inhibit the AMPK-related kinase SNARK in vitro
Source: Oncotarget. 2016 Sep 2;7(46):74987–99. doi: 10.18632/oncotarget.11820 (PMC5342717; doi:10.18632/oncotarget.11820)
Supplement: Supplementary file 1 [file oncotarget-07-74987-s001.pdf]

Anti-hepatocellular carcinoma properties of the anti-alcoholism drug disulfiram discovered to enzymatically inhibit the AMPK-related kinase SNARK *in vitro*

SUPPLEMENTARY FIGURES AND TABLE

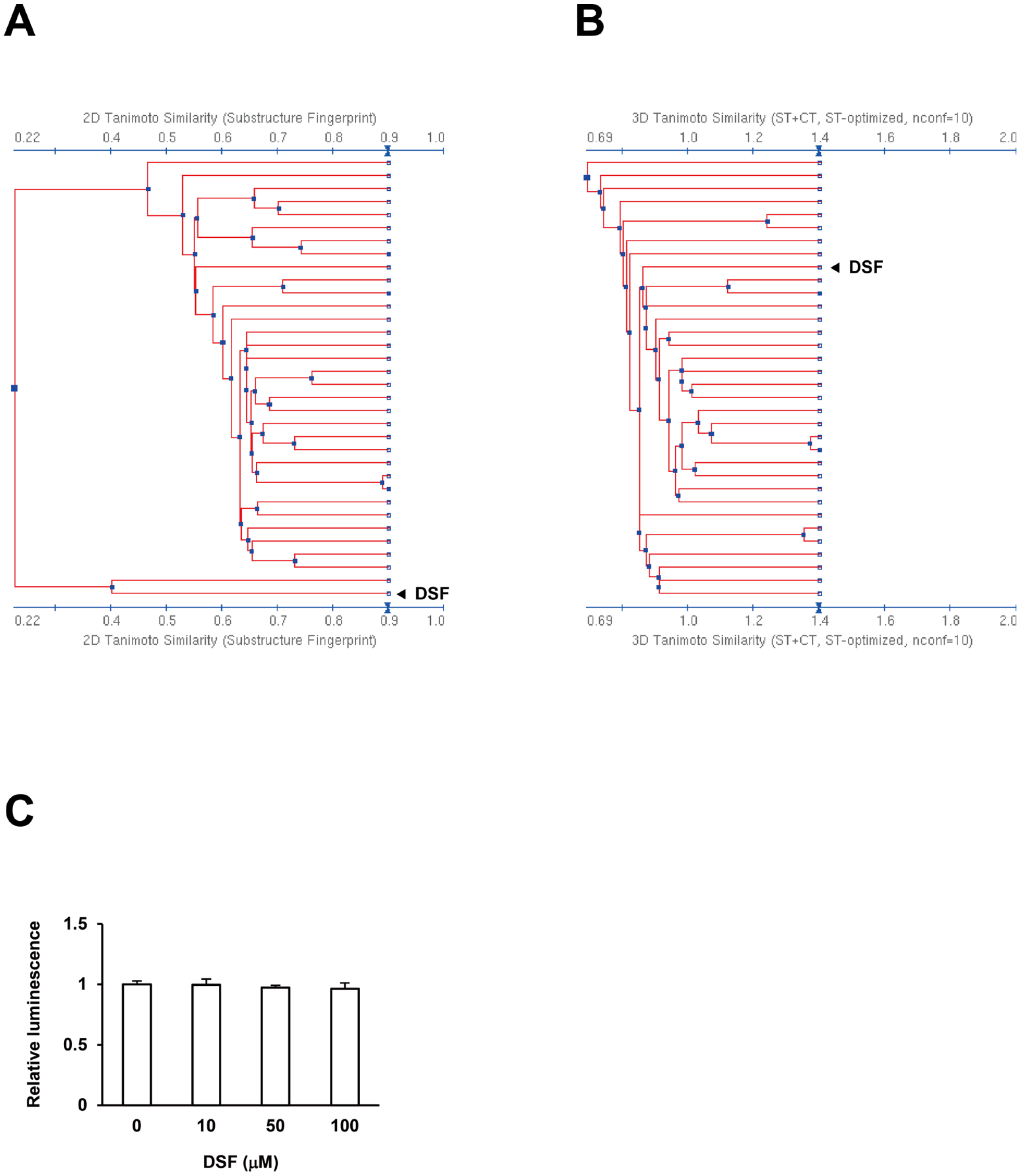

Supplementary Figure S1: Top hits in the primary screen. Hits with inhibition more than 50% were clustered based on the 2D **A.** and 3D **B.** structures in PubChem; DSF is indicated by arrowhead. **C.** The *in vitro* kinase assay was performed without CHKtide to monitor the basal level of luciferase activity itself, in the presence of 10, 50, and 100 μM DSF.

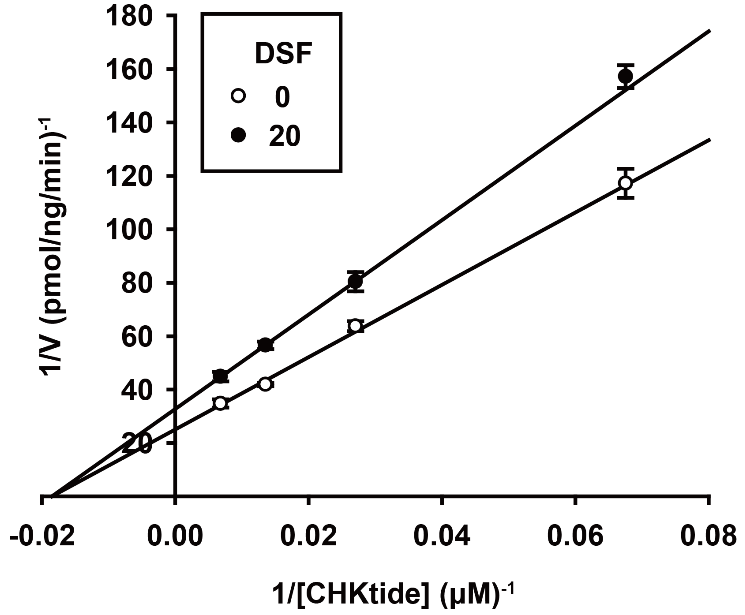

Supplementary Figure S2: Enzymatic kinetics of SNARK kinase inhibition by DSF after preincubation. After the incubation of SNARK protein with DSF at 20 μM for 1h, the *in vitro* kinase assay was performed similarly to Figure 1D. Subsequently the effects of DSF on enzymatic kinetics of SNARK were calculated similarly to Figure 1B, yielding Lineweaver-Burk plots.

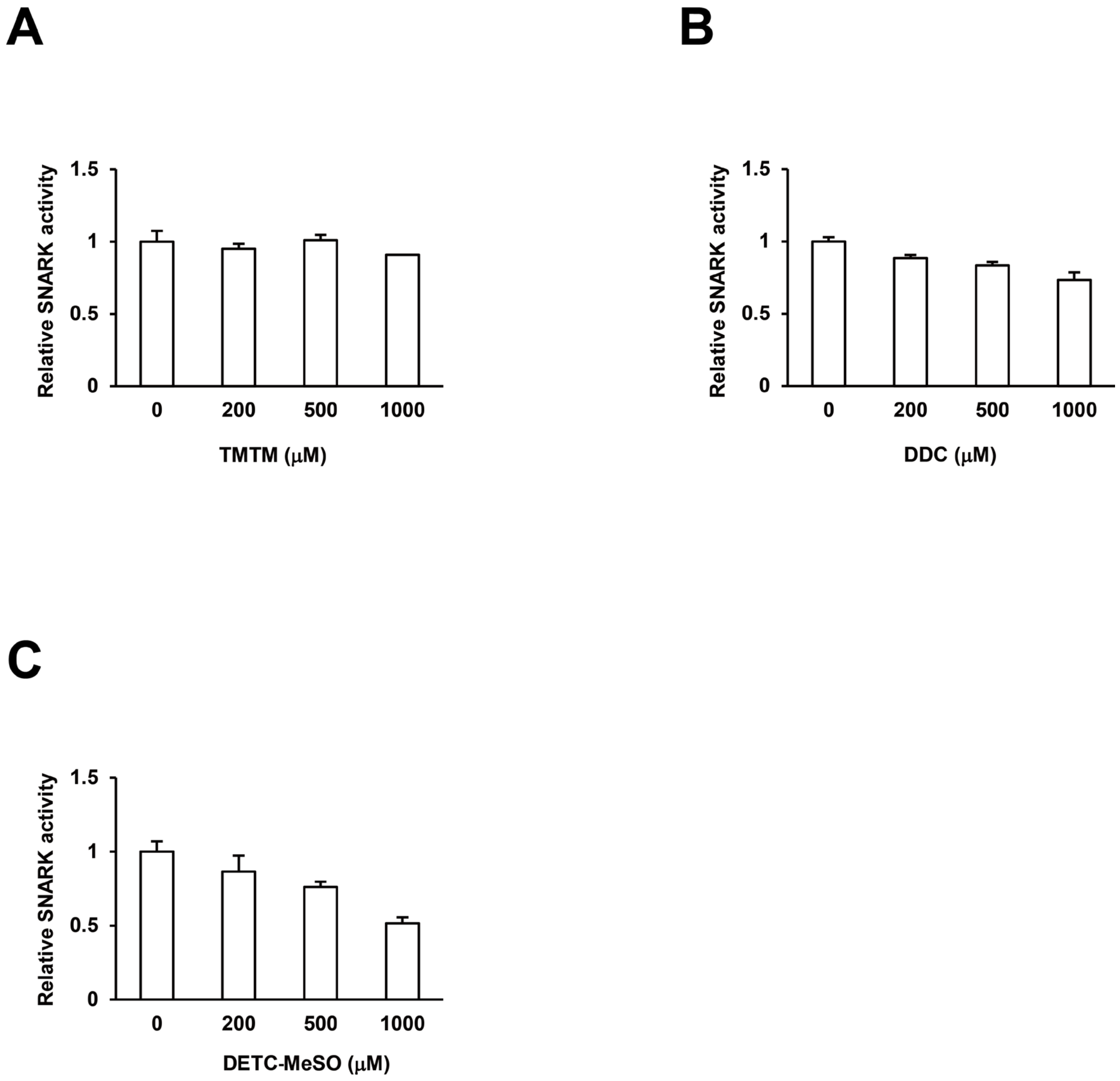

Supplementary Figure S3: Effects of DSF analogs at higher concentrations on SNARK kinase activity *in vitro*. The *in vitro* SNARK kinase assay was performed similarly to Figure 3I, J, and L, in the presence of 200, 500, and 1000 μM TMTM **A.** DDC **B.** and DETC-MeSO **C.**

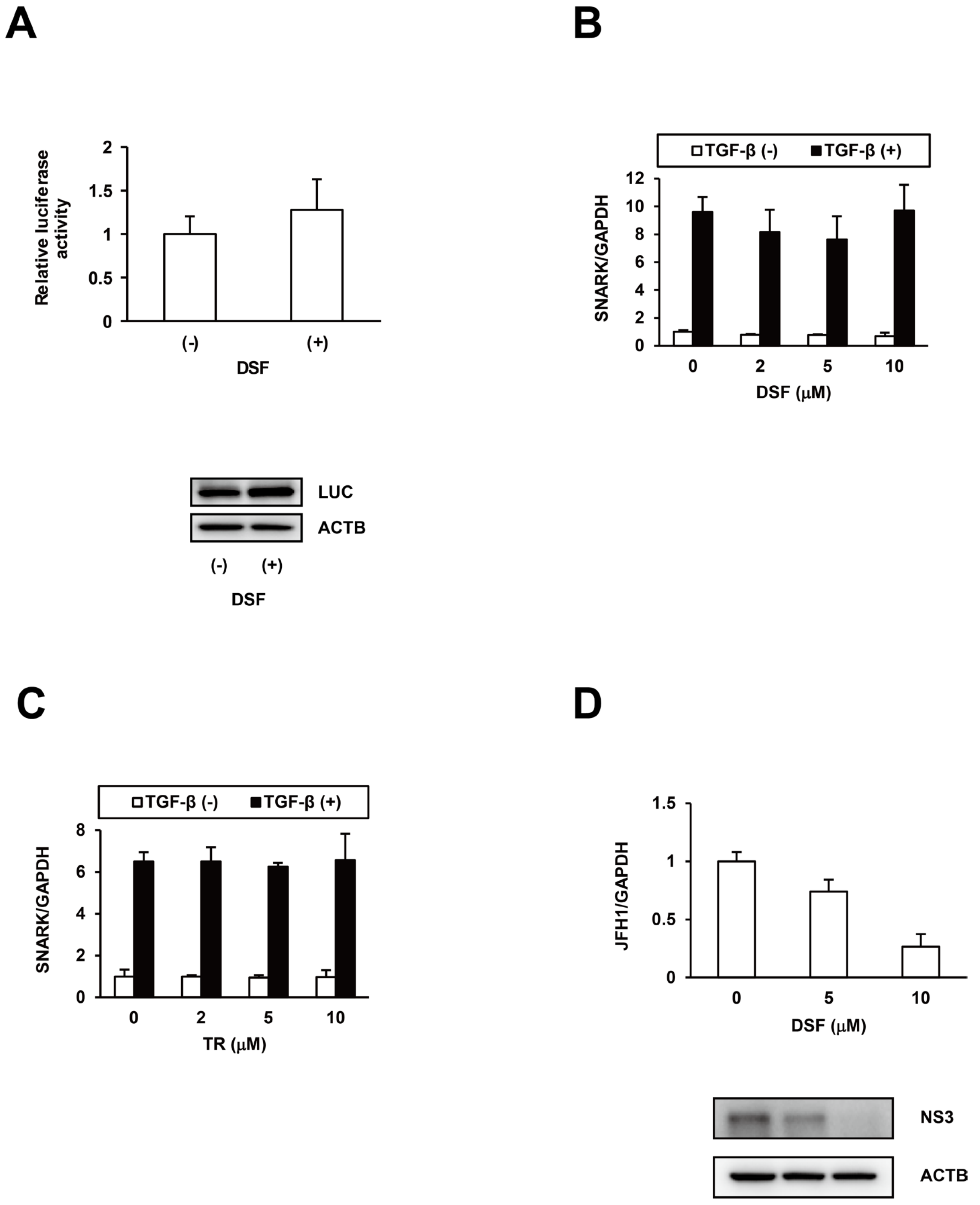

Supplementary Figure S4: Effects of DSF in cell culture. A. HepG2 cells were transfected with pEBMP-Luc, followed by the treatment with DSF at 15 μM 24 h later. On the next day the cells were lysed, and the activity and expression of firefly luciferase were measured by luciferase assay and western blotting, respectively. The protein expression of ACTB was also detected similarly. HepG2 cells were treated with DSF **B.** and TR **C.** at 2, 5, and 10 μM in the absence or presence of TGF-β at 10 ng/mL for 48h and SNARK mRNA levels were quantified by qRT-PCR with normalization to *GAPDH*, similarly to Figure 4G. **D.** Huh7.5.1 cells infected with JFH1 for 24h were treated with 5 and 10 μM DSF for 48h, followed by the quantification of JFH1 RNA levels by qRT-PCR with normalization to *GAPDH* and the detection of HCV NS3 and ACTB by western blotting.

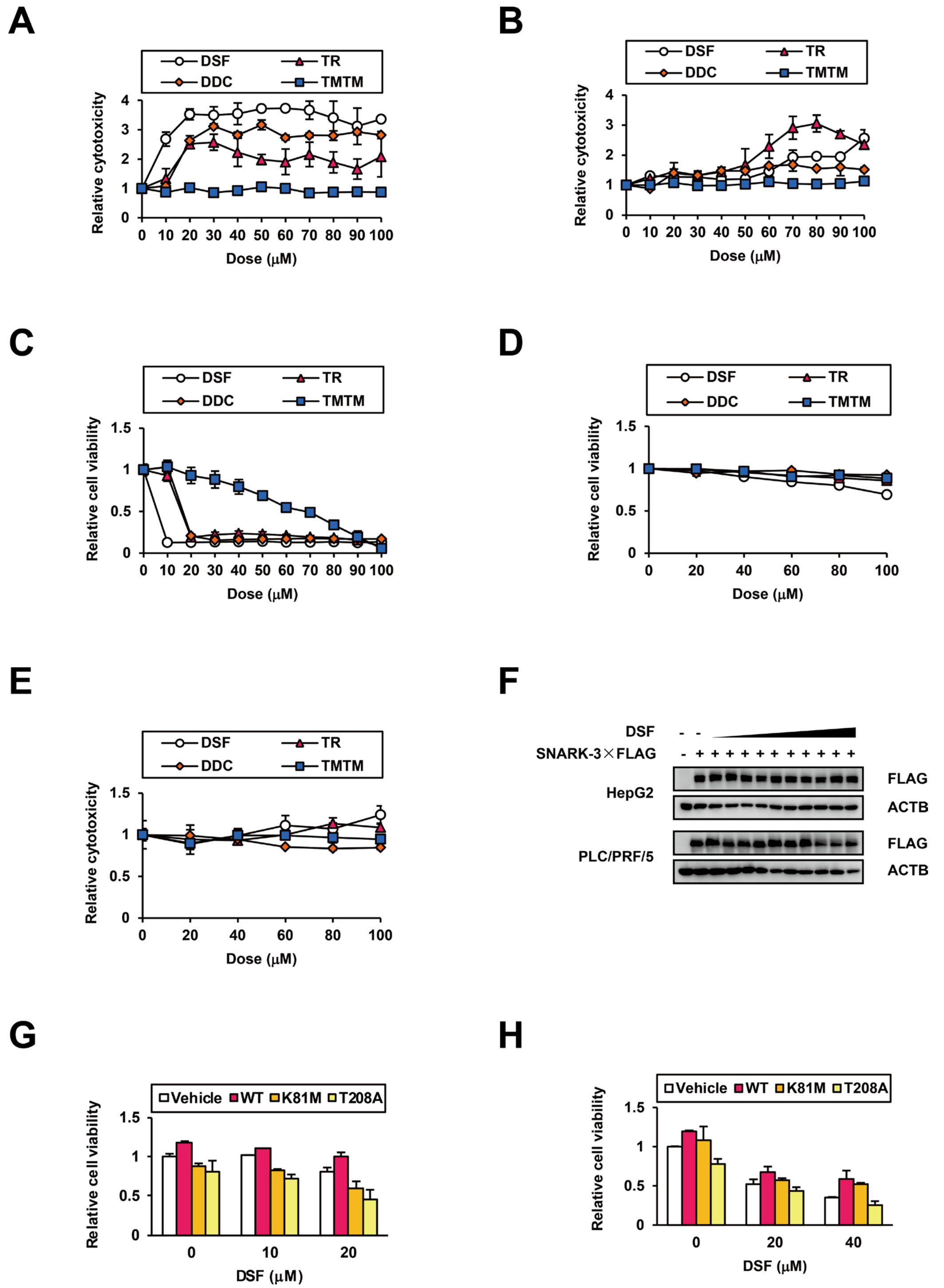

Supplementary Figure S5: Selective anti-HCC cell activities of DSF via SNARK function. HepG2 **A.** PLC/PRF/5 **B.** and Huh7 **C.** cells were treated with DSF, TR, TMTM, and DDC at the indicated doses for 24 hours and the relative cytotoxicity and cell viability to untreated control cells were measured by LDH release and CCK-8 assays. PXB cells were treated with with DSF, TR, TMTM, and DDC at the indicated doses for 24 hours and the relative cell viability and the relative cytotoxicity to untreated control cells were measured by CCK-8 **D.** and LDH release **E.** assays, respectively. **F.** After the transfection of pEBMP-SNARK-3×FL and the treatment with DSF at 10-100 μM in HepG2 and PLC/PRF/5 cells as in Figure 5C and D, FLAG and ACTB were detected by western blotting. Also similarly to Figure 5C and D, HepG2 **G.** and PLC/PRF/5 **H.** cells were transfected with pEBMulti-Puro (Vehicle), pEBMP-SNARK-3×FL (WT), pEBMP-SNARK(K81M)-3×FL, or pEBMP-SNARK(T208A)-3×FL, followed by DSF treatment at the indicated concentrations 48h later. On the next day the relative cell viabilities to the control samples untreated and transfected with pEBMulti-Puro were measured.

Supplementary Table S1: Characters of DSF analogs and SNARK inhibition

Compound	IC50 (μM)	Disulfide bond	Inhibition mode	Ki (μM)
DSF	43.7	(+)	Noncompetitive	65.7 ± 6.09*
				64.3 ± 5.17†
TR	42.0	(+)	Noncompetitive	79.0 ± 5.25*
				138.0 ± 21.3†
TMTM	> 1000	(-)	N/A	N/A
DDC	> 1000	(-)	N/A	N/A
DETC-MeSO	> 1000	(-)	N/A	N/A

*For ATP.

†For CHKtide.
